# Hydrops Fetalis due to Kell Alloimmunization: A Perinatal Approach to a Rare Case

**DOI:** 10.5505/tjh.2012.37801

**Published:** 2012-03-05

**Authors:** Arzu Akdağ, Ömer Erdeve, Nurdan Uraş, Yavuz Şimşek, Uğur Dilmen

**Affiliations:** 1 Zekai Tahir Burak Maternity and Teaching Hospital, Ankara, Turkey

**Keywords:** Hydrops fetalis, Anemia, Kell alloimmunization, newborn

## Abstract

**Objective:** While routine administration of rhesus (Rh) immunoglobulin has significantly reduced the incidence of Rh alloimmunization, maternal alloimmunization to other red cell antigens remains a contributor to perinatal morbidity and mortality. Although the Kell antigen is seen on the red cells of only 9% of the general population, attention to Kell antibodies continues to increase.

**Case Report:** A case of fetal hydrops was sonographically detected at 30 weeks of gestation. Antenatal tests to evaluate the fetus’s condition clearly showed that the level of hemolytic disease was critical and the baby was delivered prematurely due to fetal distress. The combination of anemia, reticulocytopenia, hydrops fetalis, and a positive indirect Coombs test suggested Kell isoimmunization. The baby was successfully treated with exchange transfusion of Kellnegative packed red cells, and was discharged on postnatal d 30.

**Conclusion:** The presented case of hydrops fetalis was due Kell alloimmunization that was detected during the postnatal period, and thus we plan to discuss the perinatal approach to Kell immunization.

## Abstract (tr)

**Amaç:** Günümüzde Rhesus (Rh) immunoglobulinin yaygın olarak uygulanması RhD alloimmünizasyon insidansında azalmaya neden olurken RhD dışındaki eritrosit antijenleri ile maternal alloimmunizasyon perinatal morbidite ve mortaliteye yol açmaya devam etmektedir. Kell antijeni toplumda yalnızca %9 oranında bulunmasına rağmen bu antijene bağlı alloimmunizasyon günümüzde ön plana çıkmaya başlamıştır.

**Olgu Sunumu:** Hastamız 30. gebelik haftasında antenatal fetal hidrops tanısı almıştı. Fetal durumu değerlendirmeye yönelik antenatal testler kritik düzeyde hemolitik hastalığı gösterdi, ancak hasta fetal distres nedeniyle prematüre olarak doğurtuldu. Anemi, retikülositopeni, hidrops fetalis ve pozitif indirekt Coombs testi Kell izoimmunizasyonunu düşündürdü. Postnatal dönemde Kell-negatif eritrosit süspansiyonu ile kan değişimi uygulanan hasta postnatal 30.gün şifa ile taburcu edildi.

**Sonuç:** Bu makalede, Kell izoimmunizasyonuna bağlı geliştiğini postnatal dönemde saptadığımız bir hidrops fetalis olgusu sunularak Kell immunizasyonuna perinatal yaklaşımın tartışılması planlandı. Bu durum fetusun antijen negatif uygun kan ile başarılı bir şekilde tedavi edilmesine olanak sağlayacaktır.

## INTRODUCTION

Hydrops fetalis is the excessive accumulation of fluid in the subcutaneous tissues and serous cavities of fetuses and neonates. The first cases described were associated with Rhesus (Rh) alloimmunization. While routine administration of Rh immunoglobulin has significantly reduced the incidence of this type of alloimmunization, maternal alloimmunization to other red cell antigens remains a contributor to perinatal morbidity and mortality. Although the Kell antigen is seen on the red cells of only 9% of the general population, attention to Kell antibodies continues to increase. As a possible factor associated with fetal anemia in the case of Kell alloimmunization is suppression of erythropoiesis, reticulocyte and normoblast counts are inappropriately low for the degree of fetal anemia in fetuses and neonates. Herein we describe a preterm neonate with hydrops fetalis due to Kell isoimmunization that was detected during the postnatal period, and discuss the perinatal approach to this rare condition.

## CASE REPORTS

A male neonate was delivered via cesarean section after 32 weeks and 5 days of gestation to a 24-year-old gravida 3, para 2 mother. The patient’s 1st and 5th min APGAR scores were 3 and 6, respectively. The newborn required intubation and ventilation support due to respiratory distress that developed after immediately following birth and was admitted to the neonatal intensive care unit. Routine ultrasonographic examination at 30 weeks of gestation showed fetal ascites and cardiomegaly, and fetal echocardiography confirmed myocardial hypertrophy. Doppler measurement of the middle cerebral artery (MCA) was performed for the prediction of fetal anemia and the peak systolic velocity (PSV) was significantly elevated (60 cm s–^1^).

Several tests were performed to determine the etiology of the fetal anemia during the pregnancy. The mother’s blood group was AB Rh(+); therefore, Rh and ABO incompatibility were eliminated as the cause of hydrops. IgM antibody against parvovirus B19 (the etiological agent of anemia) was negative. The mother’s obstetric history included normal full-term delivery of a healthy male 4 years earlier, and dilation and curettage (D&C) two years ago. The mother had never received a blood transfusion;

her and her family’s medical history were otherwise unremarkable.

Physical examination of the neonate neonate immediately following birth showed marked pallor and gross skin edema. He weighed 2600 g, his respiratory rate was 72 breaths min–^1^ with retractions, and vesicular breath sounds were audible. A grade 2/6 systolic murmur was heard and abdominal distention was noted due to massive ascites. The liver and spleen were palpable 2 cm and 1 cm below the costal margins, respectively. Initial complete blood count findings were as follows: white blood cell count: 12,600/ mm^3^; hemoglobin level: 4 g /dL1; hematocrit: 12%; platelet count: 240,000 /mm^3^; reticulocyte count: 2%. Biochemical analyses performed at birth showed that the albumin level was 2.45 g /dL1 (normal range: 3.5-5 g/ dL1). We investigated the etiology of anemia. Sickle cell test, hemoglobin electrophoresis results, and the glucose-6-phosphate dehydrogenase enzyme level were normal. Echocardiography was normal.

The combination of anemia, reticulocytopenia, and hydrops fetalis was thought to be consistent with Kell isoimmunization. A special request was sent to the blood bank to screen for uncommon rare blood groups and non-D antibodies. The patient’s Kell antigen was positive, his mother’s was negative, and the maternal serum indirect Coombs test was positive; therefore, the diagnosis of Kell isoimmunization was confirmed. Due to severe anemia, the patient underwent exchange transfusion with ABpositive, Kell-negative packed red cells on postnatal d 1; he received transfusion of an additional unit of packed red cells with compatible AB-positive and Kell-negative cross matching on postnatal d 3. Throughout the newborn’s hospitalization hyperbilirubinemia was not observed. The patient was then managed conservatively and was discharged in stable condition on postnatal d 30.

## DISCUSSION

More than 50 different red-cell antigens are associated with hemolytic disease of the fetus and newborn (HDFN), but most cases of severe fetal disease are caused by anti- RhD, anti-RhC, and anti-Kell (K1) [1]. Alloimmunization to RhD erythrocyte antigen has historically been the most common etiology of severe HDFN; however, this type of alloimmunization is becoming rare in developed countries as a result of routine prophylaxis with anti-D IgG. Nonetheless, HDFN continues to cause fetal morbidity and mortality and attention to other more rare blood group antibodies for which immune prophylaxis is unavailable continues to increase. In the presented patient hydrops fetalis due to severe anemia was associated with maternal Kell alloimmunization.

Maternal anti-K is the second most common alloantibody (anti-D is the first) and accounts for 10%-15% of all patients with antibody-mediated severe hemolytic anemia [2]. Currently, the rate of hemolytic disease related to Kell antibodies is increasing, but the cause for this increase remains unknown. Previous blood transfusion is thought to be an etiological cause of their sensitization, as 66% of women with Kell antibodies have a history of transfusion in the absence of routine Kell antibody screening prior to transfusion [3]. At least 8 different antigens of the 24-member Kell red-cell antigen system have been associated with HDFN. The most common of these are Kell (k, K1) and Cellano (k, K2). The K1 antigen is found on the red cells of 9% of the general population, and virtually all antigen-positive individuals being heterozygous. These gene frequencies are associated with a 5% risk of an affected fetus in Kell-alloimmunized pregnancies. One study reported that maternal anti-K was observed in 459 pregnancies; 20 fetuses among the 459 pregnancies with maternal anti-K were affected. In all, 4 of the 20 affected fetuses with hydrops died—3 in utero and 1 during the neonatal period. [4]. McKenna et al. studied 156 pregnancies in 134 women with anti-K1 antibodies. In all, 8 infants (5%) were severely affected, 6 of which received intrauterine transfusion; there were 3 fetal deaths. An additional 13 infants (8%) were affected with mild HDFN [3].

The clinical features of anti-K HDFN differ from those of the classical form of the disease caused by anti-D. As the severity of intrauterine disease may not correlate with maternal serum antibody titers, the utility of antibody titers in monitoring K-antigen-sensitized pregnancies is limited [4,5]. A critical maternal indirect Coombs titer of ≥8 is considered the threshold for suspicion of fetal anemia and close monitoring of these fetuses is recommended. Prediction of the severity of anti-K disease based on amniotic fluid bilirubin levels obtained from serial amniocentesis is not possible because anti-K HDFN is associated with lower concentrations of amniotic fluid bilirubin than anti- D HDFN [6,7]. Postnatal hyperbilirubinemia is not significant in newborns with hemolytic disease caused by anti-K.

There are fewer numbers of circulating reticulocytes and normoblasts in fetuses and newborns with anti-K disease than in fetuses with anti-D HDFN [7]. Reticulocytosis and erythroblastosis occur at lower rates in fetuses with anti-K disease than in those with anti-D HDFN [7].

These observations suggest that the pathogenesis of HDFN caused by anti-K differs from that due to anti-D. The body of in vitro and in vivo evidence suggests that fetal anemia in cases of Kell sensitization is secondary to 2 mechanisms: red cell destruction (less common) and suppression of erythropoiesis (more common) [7,8]. This also leads to fetal anemia in the absence of a concomitant increase in bilirubin breakdown products, thus leading to relatively normal delta OD450 values. In the presented patient reticulocyte and normoblast counts were low, which is consistent with Kell isoimmunization, whereas maternal antibody levels were unexpectedly very high. The presented patient’s negative direct Coombs test indicated that erythroid suppression, rather than hemolysis was the predominant mechanism responsible for fetal anemia secondary to maternal Kell alloimmunization.

Serial PSV measurement in the fetal MCA can be used to assess fetal anemia after 16-18 weeks of gestation; a value >1.5 MoMs indicates the need for cordocentesis and possible intrauterine blood transfusion with Kell-negative packed red cells [9,10]. In the presented case MCA Doppler measurement showed that the PSV was critically high level; however, before further testing could be performed the patient was delivered prematurely due to fetal distress. The neonate was treated postnatally with exchange transfusion and neonatal anemia significantly improved following transfusion with Kell-negative blood.

## CONCLUSION

Anti-Kell alloimmunization is a potentially serious cause of fetal anemia. In such cases antenatal treatment may help prevent fetal and neonatal complications. Furthermore, we highlight the importance of irregular antibody screening for women with significant obstetric history and fetal hydrops. Lastly, use of Kell-negative blood for transfusion in all females during childhood and throughout their reproductive years should further reduce the incidence of Kell-alloimmunization.

## CONFLICT OF INTEREST STATEMENT

The authors of this paper have no conflicts of interest, including specific financial interests, relationships, and/ or affiliations relevant to the subject matter or materials included.
